# Customized chitooligosaccharide production—controlling their length *via* engineering of rhizobial chitin synthases and the choice of expression system

**DOI:** 10.3389/fbioe.2022.1073447

**Published:** 2022-12-14

**Authors:** Rita Weyer, Margareta J. Hellmann, Stefanie N. Hamer-Timmermann, Ratna Singh, Bruno M. Moerschbacher

**Affiliations:** Institute for Biology and Biotechnology of Plants, University of Münster, Münster, Germany

**Keywords:** chitooligosaccharides, chitooligosaccharide production, chitin synthase, chitin oligomer, rhizobia, cos, protein engineering, nodc

## Abstract

Chitooligosaccharides (COS) have attracted attention from industry and academia in various fields due to their diverse bioactivities. However, their conventional chemical production is environmentally unfriendly and in addition, defined and pure molecules are both scarce and expensive. A promising alternative is the *in vivo* synthesis of desired COS in microbial platforms with specific chitin synthases enabling a more sustainable production. Hence, we examined the whole cell factory approach with two well-established microorganisms—*Escherichia coli* and *Corynebacterium glutamicum*—to produce defined COS with the chitin synthase NodC from *Rhizobium* sp. GRH2. Moreover, based on an *in silico* model of the synthase, two amino acids potentially relevant for COS length were identified and mutated to direct the production. Experimental validation showed the influence of the expression system, the mutations, and their combination on COS length, steering the production from originally pentamers towards tetramers or hexamers, the latter virtually pure. Possible explanations are given by molecular dynamics simulations. These findings pave the way for a better understanding of chitin synthases, thus allowing a more targeted production of defined COS. This will, in turn, at first allow better research of COS’ bioactivities, and subsequently enable sustainable large-scale production of oligomers.

## 1 Introduction

Growing environmental awareness raises interest in renewable natural compounds such as the highly versatile, biocompatible and biodegradable chitin oligomers and partially acetylated chitosan oligosaccharides (COS and paCOS, respectively) (Liaqat and Eltem, 2018; Schmitz et al., 2019). Their uses include i.a. various biomedical (Yin et al., 2017; Santos-Moriano et al., 2018; Naveed et al., 2019; Kumar and Kumar, 2020) as well as plant growth promoting and strengthening (Winkler et al., 2017; Zhang et al., 2019) applications, but the list of current and potential uses is steadily growing (Yuan et al., 2019; Zhao et al., 2019).

Currently, a majority of these multifunctional biologics is generated from marine wastes, namely crab and shrimp shells, out of which chitin—a polymer of β-1,4-linked *N*-acetyl-d-glucosamine units (GlcNAc)—is thermo-chemically extracted. It can then be partially deacetylated by alkaline treatment and optionally partially depolymerized by acid hydrolysis to yield chitosan polymers or paCOS, respectively (Kaczmarek et al., 2019; Schmitz et al., 2019). This conventional process is convenient, efficient and low cost, but also very energy-consuming, and it produces considerable amounts of hazardous waste (Kaczmarek et al., 2019). Moreover, it is difficult to control and yields mixtures of varying length (degree of polymerization, DP), degree of acetylation (DA) (Kaczmarek et al., 2019; Yuan et al., 2019), and a more or less random pattern of acetylation (PA) (Trombotto et al., 2008; Weinhold et al., 2009; Bonin et al., 2020). Importantly, partial hydrolysis of chitin to produce fully acetylated COS is difficult to control as it easily proceeds to monomer production and in addition, easily leads to partial deacetylation, yielding paCOS instead of COS. This is problematic as DP, DA, and PA are key parameters determining bioactivities of COS and paCOS (Zou et al., 2017; Basa et al., 2020; Cord-Landwehr et al., 2020). Furthermore, removal of sometimes toxic by-products is time-consuming and expensive (Lieder et al., 2013; Kaczmarek et al., 2019; Yuan et al., 2019; Kumar and Kumar, 2020). Alternatives, such as the biotechnological extraction of chitin or the enzymatic or physical depolymerization are less efficient and more costly or energy-intensive while giving low yields (Berezina, 2016; Kaczmarek et al., 2019). Consequently, numerous studies conducted with conventional, chemically produced COS/paCOS suffer from the use of poorly characterized molecules, mixtures, and/or impurities (Guan et al., 2019; Bonin et al., 2020).

For biological activities, the oligomer size is crucial (Yamada et al., 1993; Wu et al., 2015), with COS/paCOS of DP 4–7 being particularly interesting: Hexamers and heptamers are outstanding in different fields (Lieder et al., 2012; Zou et al., 2017); one example is the induction of plant defense responses which works best with COS of DP 6 (Shi et al., 2019) and DP 7 (Hadwiger, 2013; Gubaeva et al., 2018). In addition to DP, the DA and PA are likely to play crucial roles in the bioactivities of paCOS (Gubaeva et al., 2018; Basa et al., 2020; Bonin et al., 2020; Cord-Landwehr et al., 2020). The demand for defined and pure COS and paCOS to elucidate structure-function relationships and implement findings on a larger scale is, thus, high (Das et al., 2015; Guan et al., 2019). However, the production of pure paCOS with defined acetylation patterns is challenging, especially in larger amounts than can be obtained by chemical synthesis (Tyrikos-Ergas et al., 2021). One approach is the use of purified chitin deacetylases for the defined de- or *N*-acetylation of fully acetylated or deacetylated oligomers, respectively. This approach is currently only possible up to the size of pentamers (Hembach et al., 2017; Bonin et al., 2020), and, importantly, requires the use of DP-pure starting materials. An alternative approach is the transglycosylation of smaller COS/paCOS using purified chitinases or their biotechnologically optimized mutants (Alsina et al., 2018, 2019, 2021; Madhuprakash et al., 2018). Using this approach, PA-specific products of up to DP 10 have been synthesized, but the product range is rather limited and the reaction yields mixtures of products, requiring downstream separation (Harmsen et al., 2020).

Both of the enzymatic approaches require the use of pure COS and/or paCOS as starting materials. A promising solution for their large-scale synthesis could be the environment-friendly production of desired COS in a whole cell factory approach: heterologous expression of specific chitin synthases in well-established microbial production platforms could supply the demand for defined and pure oligomers even in large quantities at low cost (Samain et al., 1997; Naqvi and Moerschbacher, 2017). Promising candidates to produce COS with different, rather uniform DP are rhizobial NodC chitin synthases, such as from *Sinorhizobium (Ensifer) meliloti* (NodC_Sm) producing tetramers when expressed in *E. coli* (Lerouge et al., 1990; Kamst et al., 2000), or from *Rhizobium* sp. strain GRH2 (NodC_GRH2) that synthesizes pentamers and hexamers when expressed in *Rhizobium* (Lopez-Lara et al., 1995). Dorfmueller et al. have presented an analysis of NodC_Sm’s general structure and mechanism of chitin synthesis, in the process creating and thoroughly validating a structural model based on a bacterial cellulose synthase (Dorfmueller et al., 2014). In addition, the first crystal structures of two chitin synthases were recently published (Chen et al., 2022; Ren et al., 2022), providing potential alternative templates for NodC modelling.

Based on a refined NodC_GRH2 model, we identified two amino acids potentially relevant for COS length, and subsequently mutated them to increase the production of either DP 6 or 4. To assess whether the chain length of the COS is influenced by the expression host, we compared COS production in *Escherichia coli* with *Corynebacterium glutamicum*. When expressing the enzyme or its muteins*,* we observed higher yields and larger DPs in the latter host. These findings can help pave the way for customized, scalable, and cost-efficient production of well-defined COS and, based on them, well-defined paCOS.

## 2 Materials and methods

### 2.1 Bacterial strains

*Escherichia coli* strain TOP10 (Invitrogen, Darmstadt, Germany) was used for general cloning, whereas *E. coli* BL21 (DE3) (Novagen, Darmstadt, Germany) and *C. glutamicum* strain DSM20300 (Leibniz Institute DSMZ, German Collection of Microorganisms and Cell Cultures, Braunschweig, Germany) were used for COS production.

### 2.2 Vector/plasmid construction

The 9.6 kb pEKEx3: NodC_GRH2 vector was generated by introducing the chitin synthase *nodC* from *Rhizobium* sp. GRH2 (GenBank AJW76243.1) into the pEKEx3 vector (Hoffelder et al., 2010; Stäbler et al., 2011; Hamer, 2014). This 8.3 kb (9.65 kb with NodC_GRH2) shuttle vector for *E. coli* and *C. glutamicum* allows steerable expression *via* the addition of lactose or isopropyl-β-d-thiogalactopyranoside (IPTG) (de Boer et al., 1983). Its main features are the *tac* promoter which is regulated by the *lacI* repressor (de Boer et al., 1983), an optimized Shine Dalgarno sequence (AGGAGG), and a spectinomycin resistance gene (*aad9*) (LeBlanc et al., 1991).

Both the NodC_S19L and NodC_R346S mutations were introduced *via* site-directed mutagenesis with a high-fidelity polymerase and the following primer pairs: AGT​TGT​GCA​GTT​GCA​GCA​GTC​C and GAT​CAT​CGT​CAT​TGA​TGC​AAT​CAT​CAT​CAC for NodC_R346S as well as TAA​CGG​CTC​ATA​AGT​CGA​TGC​AAA​C and ACA​GCA​GAG​CGT​AGC​AGG​AG for NodC_S19L. Following the polymerase chain reaction (PCR), the fragments were separated in an agarose gel, the PCR products with the expected length were purified from the gel and subsequently ligated. Before transformation into competent *E. coli* TOP10 cells *via* heat shock, the ligation mixture underwent a DpnI digest. Next, plasmids from positive clones selected on LB plates with spectinomycin were sent for sequencing to confirm the introduction of the mutation and avoid the presence of other unwanted modifications. Finally, the correct constructs were transformed into competent *C. glutamicum* DSM20300 *via* electroporation at 2500 V, or *E. coli* BL21 *via* heat shock. All strains are preserved in glycerol stocks, more specific 1:1 concentrated broth from a 5 ml LB overnight culture:70% glycerol at −80°C.

### 2.3 Cultivation conditions, media, and sampling

*E. coli* TOP10 was grown at 37°C, the production strains at 30°C and, when in liquid culture, at 120 rpm in a Multitron standard incubator (Infors GmbH, Einsbach, Germany). Cultivation took place on LB agar (1.5% agar), in LB medium, or in Shake flask medium S (Waegeman et al., 2013) for *E. coli* and CGXII (Keilhauer et al., 1993) for *C. glutamicum*. The amount of SeO_2_ in the trace element solution for *E. coli* medium S was changed from 0.338 M to 2.7 mM. The CGXII medium was also slightly modified: it did not contain any urea and less CuSO_4_, 0.8 mM instead of 1.25 mM. All media were supplemented with the appropriate antibiotics, namely 100 μg/ml or 250 μg/ml spectinomycin for *E. coli* and for *C. glutamicum*, respectively.

For the growth experiments, a 3–6 ml LB pre-culture in 20–100 ml shake flasks (SF) with appropriate antibiotics was inoculated from a glycerol stock. After 10–13 h, the pre-culture was used to inoculate the main culture, 20 ml SF medium S or CGXII with appropriate antibiotics in a 200 ml baffled SF, to an optical density at 600 nm (OD600; Eppendorf BioPhotometer plus, Wesseling, Germany) of 0.1. Induction was performed with 0.1 mM IPTG 9–13 h later. During cultivation, 1 ml samples were taken at regular intervals for a measurement of the OD600 and subsequently stored at −20°C until further use.

### 2.4 Product analysis and quantification

#### 2.4.1 Sample preparation

Samples were thawed at room temperature. Next, 500 µl sample were transferred to 1.5 ml Eppendorf reaction tubes. Each sample underwent a thermal-mechanical treatment of 10 min at 90°C and 1,000 rpm in a BioShakeiQ (Quantifoil Instruments GmbH, Jena, Germany) for cell lysis. Cell debris was separated by centrifugation, the supernatant was directly used for LC-MS analysis.

#### 2.4.2 HPLC-ESI-MS analysis of chitin oligomers

LC-MS measurements were performed as described by Hamer et al. (2015): samples of 2 µl (1 µl for standards) were injected by an autosampler and subsequently separated by hydrophilic interaction chromatography (HILIC) using an Acquity UPLC BEH Amide column (1.7 µm, 2.1 mm × 150 mm; Waters Corporation, Milford, MA, United States) in combination with a VanGuard pre-column (1.7 µm, 2.1 mm × 35 mm; Waters Corporation, Milford, United States). With a flow rate set to 0.4 ml/min at 35°C, the samples were eluted in a 15-min method with a gradient from A and B. Eluent A consisted of 80:20 acetonitrile:water and eluent B of 20:80 acetonitrile:water. In addition, both eluents contained 10 mM NH_4_HCO_2_ and 0.1% (v/v) formic acid. The method consisted of the following steps: 0–2.5 min: isocratic, 100% A; 2.5–12.5 min: linear gradient reducing the concentration to 25% A; 12.5–13.5 min: linear gradient back to 100% A; 13.5–15 min: re-equilibration of the column with 100% A. The measurements were performed in positive mode with a target mass of 500 m/z.

#### 2.4.3 Quantification of chitooligosaccharides

**FIGURE 1:**
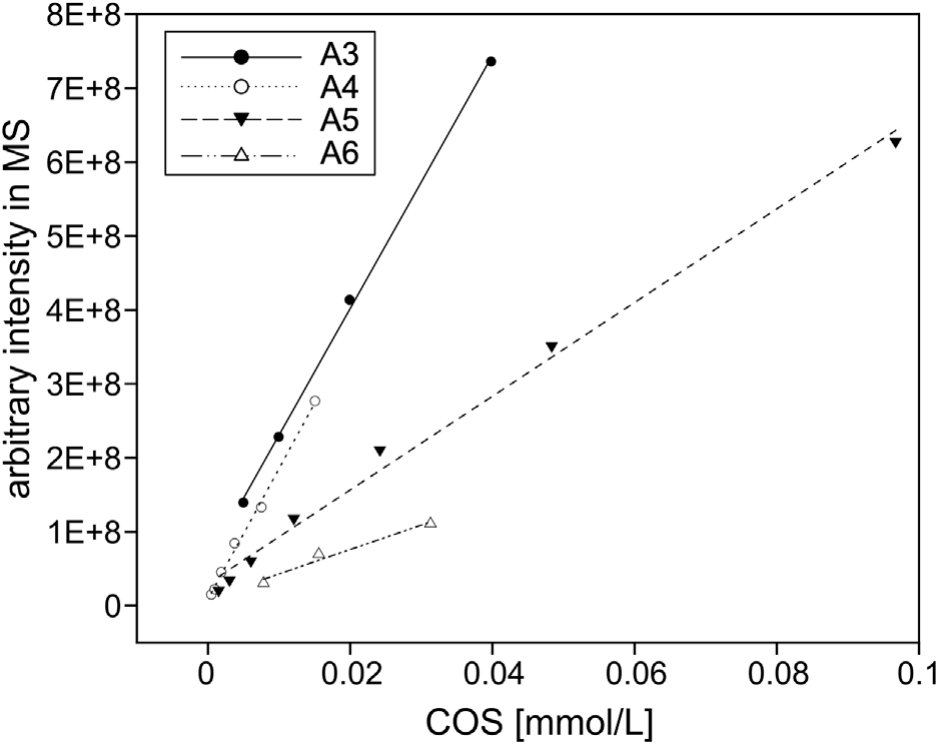
COS standards. Depicted are the arbitrary signal intensities of the ions measured by the LC-MS for up to 0.1 mM fully acetylated trimer to hexamer (A3–A6). All linear regressions feature *R* ^2^ values above 0.96 and were used to quantify COS in this study.

For the quantification of COS, the arbitrary signal intensities of the ions from the LC-MS chromatograms were converted to molar fractions and shown as a percentage share from the total amount of product. To this end, different concentrations of fully acetylated chitin oligomers from Megazyme Ltd. (Bray, Ireland) ranging from DP 3–6 (A3–A6) were injected into the UHPLC-ELSD-ESI-MS system to create linear standard curves (see Figure 1) for the conversion from arbitrary intensities into molar fractions based on the masses given in Table 1.

**TABLE 1:** Molar masses of oligomers. Molar masses of fully acetylated trimer to hexamer (A3–A6) used for conversion of LC-MS data.

Oligomer	Mass [g/mol]
A3	627.6
A4	830.4
A5	1,034.0
A6	1,237.2

### 2.5 *In silico* work

#### 2.5.1 Model generation

First, the alignment of NodC_Sm (Gene ID at NCBI: 61599275) with the bacterial cellulose synthase subunit A from *Rhodobacter sphaeroides* [BcsA; PDB code 4HG6_A (Morgan et al., 2013)] was taken from Dorfmueller et al. (2014). Next, NodC_GRH2 (UniProt: A0A0N7ARR3) was manually aligned to BcsA, analogous to NodC_Sm, and both NodC-BcsA alignments were uploaded to SWISS-MODEL for a “target-template-alignment” (Guex et al., 2009; Benkert et al., 2011; Mariani et al., 2013; Bienert et al., 2017; Waterhouse et al., 2018; Studer et al., 2020). The newly generated NodC models were aligned with the pre-translocation state of the bacterial cellulose synthase (PDB code 5EJ1) in PyMOL (Morgan et al., 2013; Schrodinger, 2020). Subsequently, a chitin hexamer built with GLYCAM (Woods Group) was inserted into the NodC models using PyMOL’s pair fitting function (Schrodinger, 2020): the position of the non-reducing end GlcNAc unit is derived from the sugar unit at the non-reducing end of the cellulose chain. For the generation of smaller COS, one or two of the reducing end units were removed.

#### 2.5.1 GROMACS simulations

The molecular dynamics simulations were performed with GROMACS (Van Der Spoel et al., 2005; Abraham et al., 2015; Pall et al., 2015) version 2019.3 as described in tutorials 2 and 5 (Lemkul, 2019) with a few changes: forcefield *gromos 54a7* (Schmid et al., 2011) was applied for all components and the parametrization of the ligand was automatically performed with the Automated Force Field Topology Builder [ATB; (Malde et al., 2011; Canzar et al., 2013; Koziara et al., 2014)]. The protein was not oriented along the *z*-axis using GROMACS commands, but the protein-ligand complex was positioned with its annotated intramembrane sections in the dipalmitoylphosphatidylcholine (DPPC) membrane using the *3-Button Editing Mouse Mode* of PyMOL (Schrodinger, 2020), before executing GROMACS command *pdb2gmx* on the protein PDB-file. While the tutorial features a 1 ns long production simulation, 100 ns simulations were performed instead. Subsequently, VMD 1.9.3 (Humphrey, W., Dalke, A. and Schulten, 1996) was used to analyze the trajectories concerning average distances between certain atoms and hydrogen bonds between selected interactions partners with a distance and angle cutoff of 3.5 Å and 35°, respectively. Based on these data and the *in vivo* results, individual frames were selected that represent the trends of the distance and hydrogen bond analyses. These frames provide a possible explanation for the observed preferences of NodC_GRH2 and NodC_Sm to produce A5 or A4, respectively.

## 3 Results

### 3.1 *In silico* structural analysis of NodC_Sm and NodC_GRH2

Based on the alignment of Dorfmueller et al. (2014), a replica of their NodC_Sm *in silico* model was generated using the subunit of a bacterial cellulose synthase (BcsA) as a template (Morgan et al., 2013). The overall structure of our NodC_Sm model closely resembles the previously published one (Dorfmueller et al., 2014), with similar positioning and length of the α-helices and β-sheets (see Supplementary Figure S1). The model (Supplementary Figure S1A) shows the typical structure of a GT-A fold glycosyltransferase, the β-α-β (single Rossmann core) fold in the cytoplasm, where several β-strands align to one continuous β-sheet (Lairson et al., 2008; Moremen and Haltiwanger, 2019).

The exact start and end of the transmembrane and cytoplasm-interfacing helices of our model (Supplementary Figure S1B) largely match the published ones (Dorfmueller et al., 2014). For the cytoplasmic secondary structures, no exact data are available, but the general structures of the models coincide. However, slight differences can be observed in the cytoplasm as there are two short regions which are recognized as α-helices in our model, but not in the published one.

**FIGURE 2:**
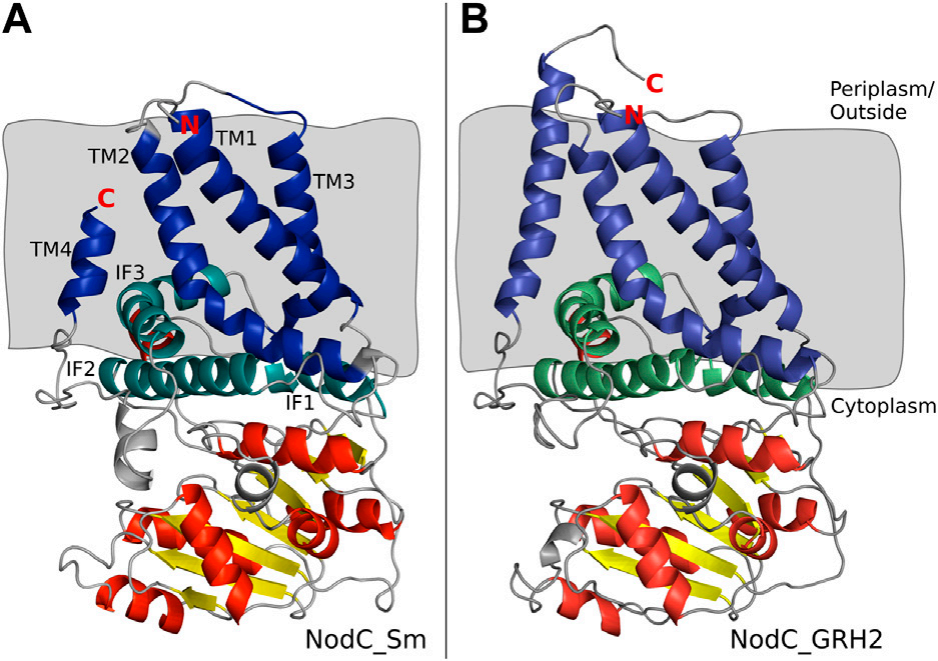
Structural model of NodC_Sm **(A)** and NodC_GRH2 **(B)** and their position within the membrane. **(A)** Blue and green highlight the exact regions identified as transmembrane domains (TM1-4, dark blue) or cytoplasmic interface-leaning helices (IF1-3, green) by Dorfmueller et al. (2014). Grey background represents the membrane. The coloring of the cytoplasmic secondary structures, the yellow β-sheets and red α-helices, as well as the indication of the N- and C-terminus with red letters are adapted from the paper (Dorfmueller et al., 2014). In comparison to the published NodC_Sm, our model shows two short extra helices in the cytoplasm (grey). **(B)** Coloring of NodC_GRH2 was made accordingly and visualizes the high similarity to NodC_Sm.

**FIGURE 3:**
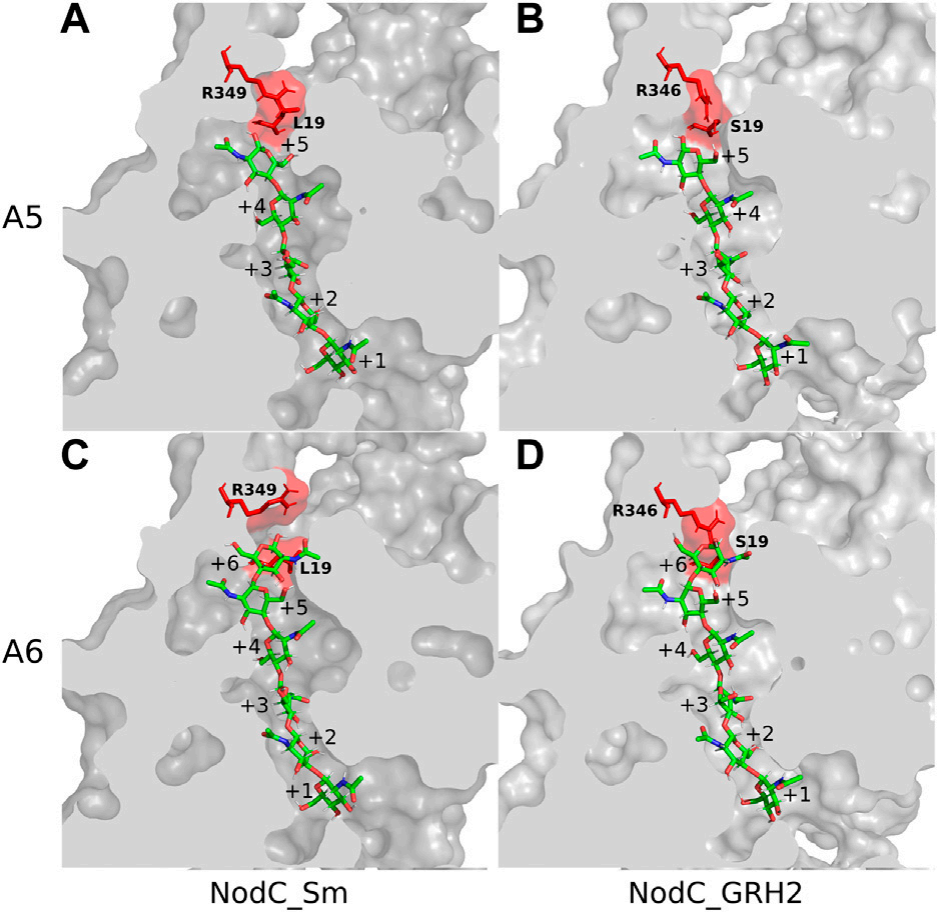
Chitin synthases NodC_GRH2 and NodC_Sm with pentamer/hexamer in their product binding sites. Depicted are the enzymes with the respective chitooligosaccharide (COS) after energy minimization. The two amino acids potentially limiting COS length are highlighted in red: R349 and L19 for NodC_Sm **(A,C)** and R346 and S19 for NodC_GRH2 **(B,D)**. Both NodC_Sm **(A)** and NodC_GRH2 **(B)** naturally produce chitin pentamer, but NodC_Sm’s main product is the tetramer. To accommodate a hexamer, bulky amino acids such as the arginine must be rotated away from the COS for NodC_Sm **(C)**, while NodC_GRH2 has more space and a sixth binding site **(D)**. The COS are shown in a stick representation, with green carbon, red oxygen, and blue nitrogen atoms.

Subsequently, the NodC_GRH2 sequence was aligned with BcsA, analogous to NodC_Sm (Dorfmueller et al., 2014), and a structural model of NodC_GRH2 was thus generated (Figure 2B). The two NodCs are not only highly similar in their sequence (70% identity of the amino acids), but also in their overall structure: The amount and positioning of the transmembrane and cytoplasmic interface-leaning α-helices are alike. Nevertheless, there are slight differences: Firstly, one of NodC_GRH2’s TM helices (TM4 analogue) is larger because a longer amino acid sequence was used for the construction of this model. Secondly, the periplasmic loop between TM1 and TM3 is longer for NodC_GRH2. The cytoplasmic regions are also highly homologous. However, while NodC_Sm has seven β-sheets, only six regions were identified as such in NodC_GRH2, where the smallest one is missing. Moreover, one region identified as a short helix for NodC_Sm was modelled as a loop in NodC_GRH2 (between IF3 and TM4). Chitin synthases catalyze the transfer of GlcNAc from uridine diphosphate-*N*-acetyl-d-glucosamine (UDP-GlcNAc) to an acceptor at the cytoplasmic site of the plasma membrane (Coutinho et al., 2003; Orlean and Funai, 2019). Thereby, the nascent chitin chain grows into a deep cavity in the enzyme (Dorfmueller et al., 2014; Orlean and Funai, 2019). In fungal chitin synthases, this cavity extends into a transmembrane tunnel which guides the growing chitin chain to the outside, but NodC is built differently (Dorfmueller et al., 2014; Orlean and Funai, 2019): both *in silico* models show a dead-end tunnel-like structure, a cleft accommodating the growing COS which opens to the cytoplasm only (Figure 3). In line with the reported product range, our and the NodC_Sm model from 2014 have five product binding subsites, which are limited by two amino acids protruding into the channel, namely arginine at position 349 (R349) and leucine at position 19 (L19) (Dorfmueller et al., 2014). Analogously, the product binding site of NodC_GRH2 is limited by arginine 346 (R346) and serine 19 (S19). As shown in Figures 3A,B, both enzymes can easily accommodate a pentamer. When fitting a hexamer into the enzymes, only NodC_GRH2 can accommodate this COS without major changes (Figure 3D). In contrast, NodC_Sm had to be modified manually: the large arginine at position 349 had to be rotated to create room for the hexamer (Figure 3C).

Based on these models, two mutations were introduced in NodC_GRH2: On the one hand, the bulky arginine at position 346 was exchanged with a smaller serine to deepen the tunnel and potentially increase the amount of hexamers produced. On the other hand, the serine at position 19 was replaced with a leucine, as it is present in NodC_Sm, thus potentially decreasing the COS length.

### 3.2 Molecular dynamics simulations

In parallel to the experimental validation, molecular dynamics (MD) simulations were carried out with the wildtype enzymes NodC_GRH2 and NodC_Sm. Based on previously published experimental data (Dorfmueller et al., 2014), the enzymes were fitted into a membrane. After insertion of the chitin oligomers A4, A5, and A6, three individual 100 ns simulations per oligomer, each starting with the same frame after energy minimization, were launched.

As the triplicates did not show a consistent behavior during the 100 ns intervals, focus was put on single runs that fit the experimental data. These simulations should be understood as a tool to study and visualize potential molecular interactions explaining the experimental results and identifying further relevant amino acids.

**FIGURE 4:**
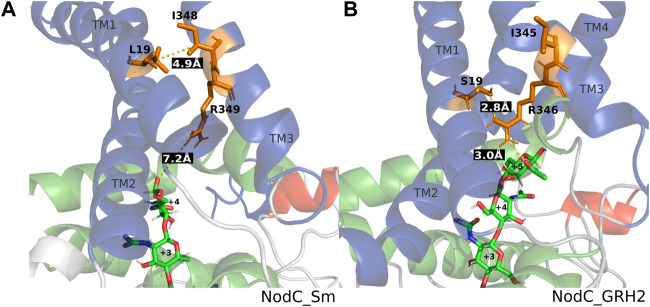
Distances within NodC_Sm **(A)** and NodC_GRH2 **(B)**. Shown are representative frames from molecular dynamics simulations. The wildtype proteins accommodate a tetramer (left) or a pentamer (right), the distances between the relevant amino acids and the COS are indicated in white font on black. Color code: blue represents transmembrane, green cytoplasmic interfacing, red cytoplasmic helices, and the chitin oligomer is shown in stick representation with green carbon, blue nitrogen, and red oxygen atoms.

Figure 4 shows selected frames of each enzyme with its natural main product, namely A4 for NodC_Sm and A5 for NodC_GRH2. These frames give a potential explanation why NodC_Sm produces shorter COS than NodC_GRH2: The simulation of NodC_GRH2 with A5 showed on average around 1.7 hydrogen bonds between S19 and R346. Correspondingly, the average distance between selected atoms of these residues is only 3.74 Å (Table 2), meaning the residues stay in proximity, like in Figure 4B. The same tendency can be observed for the hexamer, where the average distance is only slightly higher with 4.2 Å and 0.898 H-bonds. The interaction with S19 seems to avert R346 from moving further down the enzyme’s tunnel, resulting in more space for the COS product.

**TABLE 2:** Distances measured during MD simulations. Shown are the average distances from one simulation between certain amino acids [Å]. The enzymes accommodated either a chitin tetramer (A4), pentamer (A5), or hexamer (A6).

NodC_Sm			NodC_GRH2		
**Amino acids**	**A4**	**A5**	**A5**	**A6**	**Amino acids**
**L19-I348**	7.08	7.46	8.14	8.78	**S19-I345**
**L19-R349**	7.90	12.34	3.74	4.24	**S19-R346**

In contrast, the corresponding amino acids in NodC_Sm, L19 and R349, do not interact. Naturally, their side chains are unable to form hydrogen bonds, but the analysis also showed no interactions when their backbone atoms were included, regardless of the COS bound. Clearly, the average distance between selected atoms of these residues is higher than for NodC_GRH2 (7.90 Å or 12.34 Å, Table 2). Instead, a hydrophobic interaction between L19 and isoleucine at position 348 (I348) was visible in parts of the simulation, resulting in a lower average distance between selected atoms of these residues than for S19 and I345 in NodC_GRH2 (7.08 Å vs. 8.14 Å, Table 2). This interaction seems to hold together two transmembrane helices in NodC_Sm (TM1 with L19 and TM3 with I348, Figure 4A) and leaves R349 free to move further down the enzyme’s tunnel, leading to less space for COS compared to NodC_GRH2.

In contrast to NodC_Sm, the isoleucine (I345) in NodC_GRH2 is probably not involved in any interactions relevant for the COS length, as it points out of the helix, away from the product (Figure 4B). Correspondingly, regardless of the COS length, the average distance between S19 and I345 was more than 8 Å.

### 3.3 Experimental validation

After successful generation of all constructs—the pEKEx3::NodC_GRH2 wildtype (from now on referred to as NodC_GRH2), pEKEx3::NodC_GRH2_S19L (NodC_S19L) and pEKEx3::NodC_GRH2_R346S (NodC_R346S)—*E. coli* BL21 and *C. glutamicum* DSM20300 were transformed with the same constructs to reduce the vectors’ influence. In addition, equal cultivation conditions as well as the same amount of inducer were applied. Only the time of induction differed, as it was dependent on the strain-specific growth pattern: for the wildtype NodC_GRH2, the cultures’ protein production was induced after approx. 10 h of cultivation for *E. coli* and 13 h for *C. glutamicum*. Both the growth and the production of three biological replicates each are summarized in the following figures.

#### 3.3.1 Influence of the expression host

**FIGURE 5:**
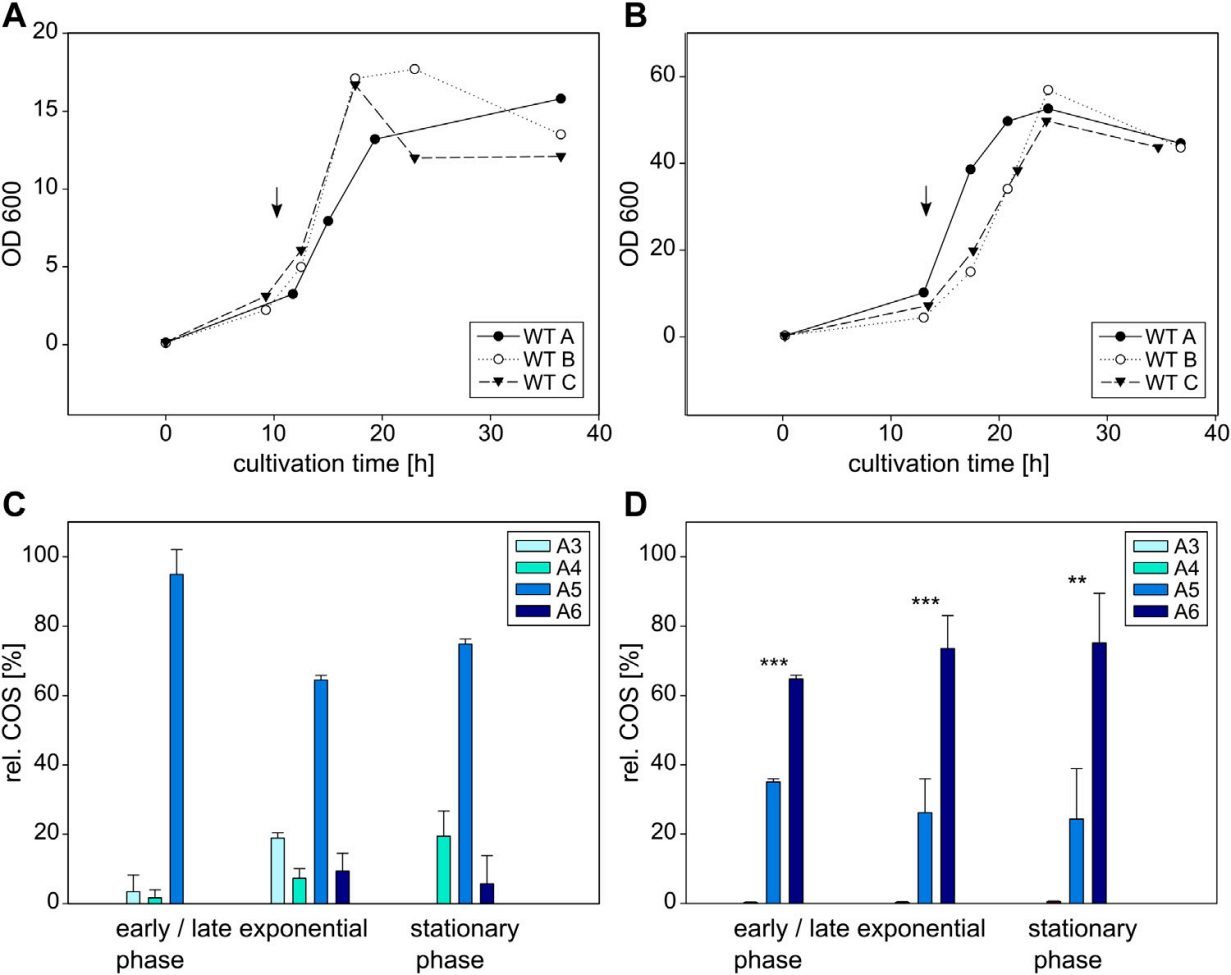
Cultivation and COS production of *E coli* BL21 (left) and *C glutamicum* (right) expressing the wildtype NodC_GRH2. Depicted is the growth of *E. coli* **(A)** and *C. glutamicum* **(B)** in triplicates as well as their product composition **(C,D)**. The time of induction is denoted with an arrow. MS signals were converted into molar fractions and are shown as shares of the total amount of product, namely the relative amount of fully acetylated trimer (A3) to hexamer (A6) for *E. coli* **(C)** and *C. glutamicum* **(D)**. The asterisks in D indicate the significance level for the difference between the product compositions in the same cultivation phase between *E. coli* **(C)** and *C. glutamicum* **(D)** determined with an independent samples *t*-test.

First, the wildtype NodC_GRH2 was heterologously expressed in both *E. coli* and *C. glutamicum*. Notably, the growth measured *via* the optical density (OD) reaches different maxima (Figures 5A,B): the values for the *E. coli* cultures (varying around 17) are clearly lower than for the *C. glutamicum* strains (ca. 50)*,* but both follow the typical pattern of lag, log, and stationary phase (Wang et al., 2015). Furthermore, all cultures were induced at the beginning of their respective exponential phase upon which COS were detected. This observation was also made for the muteins.

In *E. coli,* the main product for wildtype NodC_GRH2 throughout the whole cultivation is A5 (Figure 5C). At the beginning of the exponential phase after 13 h, it is nearly the only product detectable, accounting for 95% of all COS, with the remaining share being A3 and A4. After 18 h, 64% of COS are A5, nearly 20% are A3, with the rest being A4 (7%) and A6 (9%). In the stationary phase, pentamer is accounting for 75% of the total product, and the remaining portion consists mainly of A4 (19%) and A6 (6%).

In contrast, *C. glutamicum* expressing the same wildtype enzyme produces predominantly A6 (Figure 4D), with overall only about a quarter of the total production being A5, and A4 accounting for 1% at most, while there was no A3 at all. The share of A6 changes from 65% after 17 h at the beginning of the exponential phase, to 75% after 36 h in the stationary phase.

#### 3.3.2 Production of chitooligosaccharides in muteins

**FIGURE 6:**
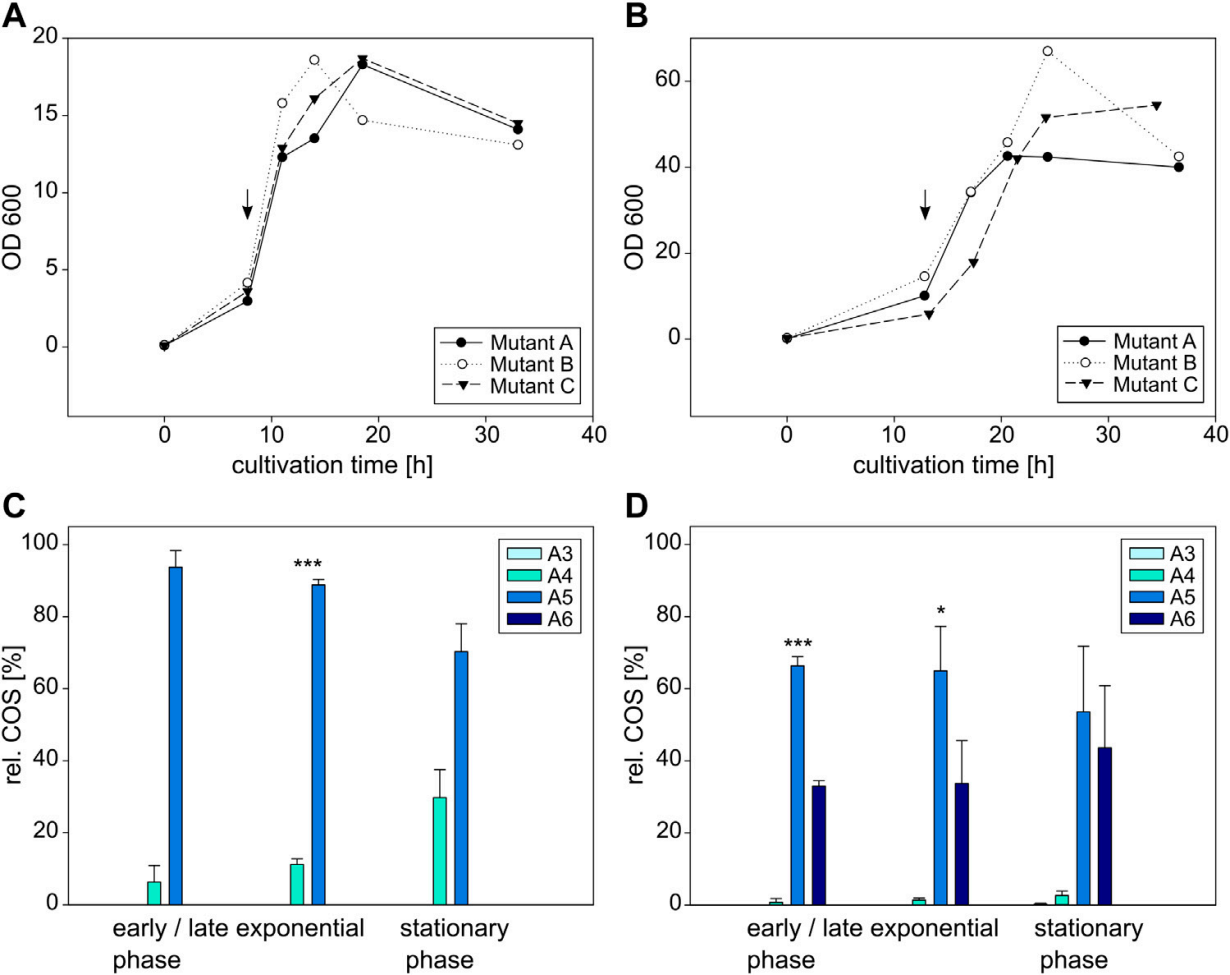
Cultivation and COS production of *E coli* BL21 (left) and *C glutamicum* (right) expressing the NodC_S19L mutein. Depicted is the growth of *E. coli* **(A)** and *C. glutamicum* **(B)** in triplicates as well as their product composition **(C,D)**. The time of induction is denoted with an arrow. MS signals were converted into molar fractions and are shown as shares of the total amount of product, namely the relative amount of fully acetylated trimer (A3) to hexamer (A6) for *E. coli* **(C)** and *C. glutamicum* **(D)**. The asterisks in C and D indicate the significance level for the difference between the product compositions in the same cultivation phase for *E. coli* between NodC_GRH2 wildtype (Figure 5C) and S19L (Figure 6C) and for *C. glutamicum* between NodC_GRH2 wildtype (Figure 5D) and S19L (Figure 6D) determined with an independent samples *t*-test.

**FIGURE 7:**
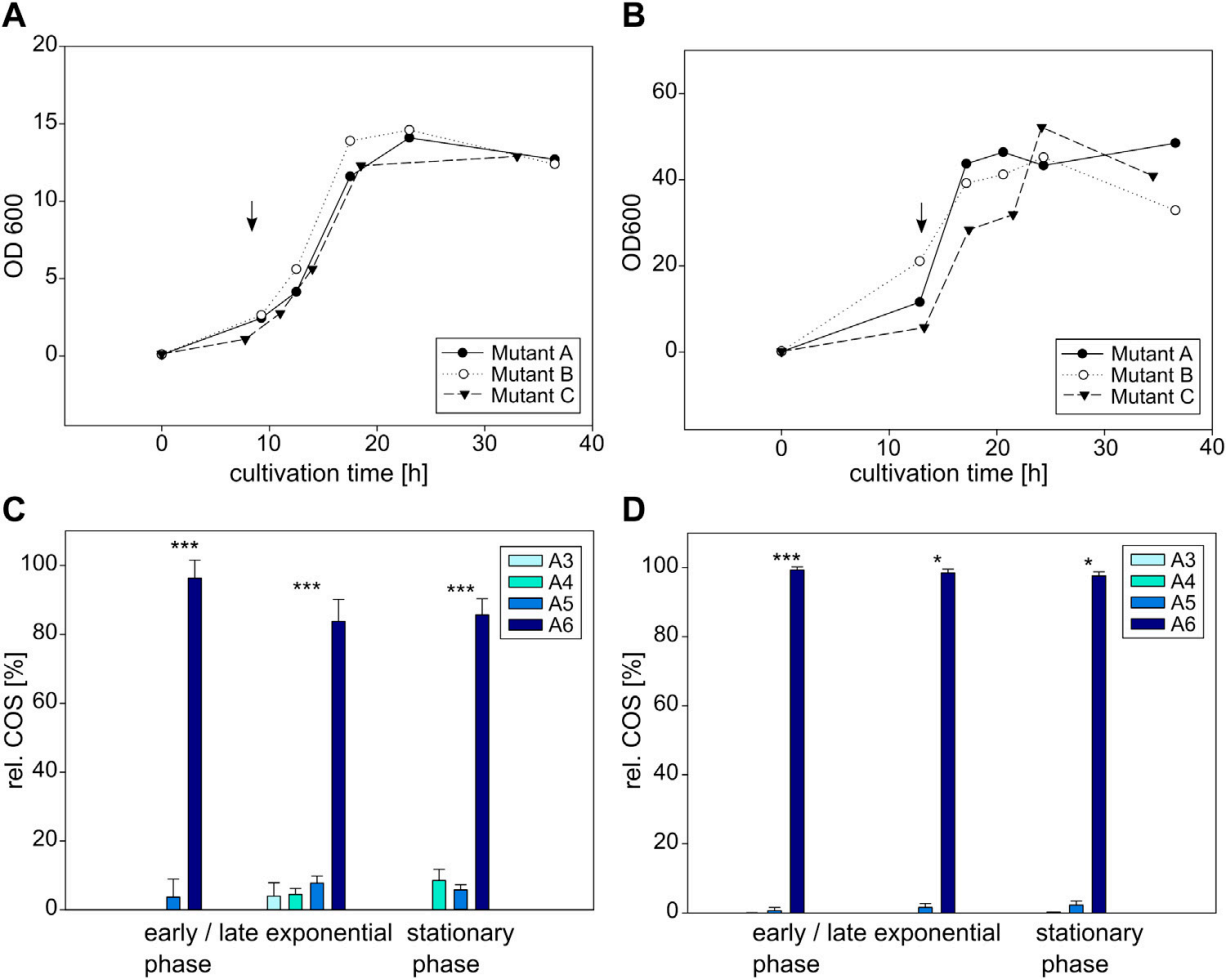
Cultivation and COS production of *E coli* BL21 (left) and *C glutamicum* (right) expressing the NodC_R346S mutein. Depicted is the growth of *E. coli* **(A)** and *C. glutamicum* **(B)** in triplicates as well as their product composition **(C,D)**. The time of induction is denoted with an arrow. MS signals were converted into molar fractions and are shown as shares of the total amount of product, namely the relative amount of fully acetylated trimer (A3) to hexamer (A6) for *E. coli* **(C)** and *C. glutamicum* **(D)**. The asterisks in C and D indicate the significance level for the difference between the product compositions in the same cultivation phase for *E. coli* between NodC_GRH2 wildtype (Figure 5C) and R346S (Figure 7C) and for *C. glutamicum* between NodC_GRH2 wildtype (Figure 5D) and R346S (Figure 7D) determined with an independent samples *t*-test.

In a second step, *E. coli* and *C. glutamicum* were cultivated expressing the NodC_S19L or the NodC_R346S muteins. Their growth and corresponding COS production are shown in the following Figures 6, 7. Like in the experiment before, the total OD600 differs strongly between *E. coli* and *C. glutamicum*, but the induction was always performed at the beginning of the exponential phase, after 9 h for *E. coli* and 13 h for *C. glutamicum*.

##### 3.3.2.1 S19L mutation: Shortening of COS

The first mutation aimed for the generation of smaller COS by introducing a leucine—like it is present in NodC_Sm—to replace the smaller serine at position 19 in NodC_GRH2.

The main oligomer detected for *E. coli* is again the pentamer, but a higher amount of tetramer is produced compared to the wildtype (Figure 6C): the product composition of *E. coli* NodC_S19L starts with 94% A5 and 6% A4 after 11 h of cultivation, shifting to 70% A5 and 30% A4 after 33 h, while the wildtype ends at only 19% A4, 75% A5, and 6% A6 (Figure 5C). Thus, with only two different COS, the products of the mutein are more uniform and, on average, a little shorter than those of the wildtype.

A similar, but more pronounced shift in product range relative to the wildtype is obtained with the same mutein in *C. glutamicum* (Figure 6D): starting with 68% A5, 33% A6, and a neglectable fraction of A4, the proportion of A5 decreases to 54%, while the shares of A6 and A4 rise to 44% and 3%, respectively. Compared to the wildtype yielding 75% A6 (Figure 5D), the main product is now the pentamer.

Clearly, in both expression hosts, the S19L mutein display a similar trend of decreased chain length compared to the wildtype.

##### 3.3.2.2 R346S mutation: Prolongation of COS

The second mutation, an exchange of arginine at position 346 with serine—like it is present in BcsA which possesses a spacious transmembrane tunnel—was performed to create room for larger oligomers.

Over the course of the whole experiment, the main product for *E. coli* expressing NodC_R346S is A6, accounting for approx. 85% of the yield, with A5 and A4 as by-products (Figure 7C). At the beginning of the exponential phase, after 12 h, 96% of the produced COS are A6, and the remaining portion is A5. Six hours later, a broader mixture was produced: the hexamer makes up for 84%, A5 for 8%, A4 and A3 for 5% and 4%, respectively. In the stationary phase, 86% are hexamer, with 9% A4 and 6% A5. Compared to the wildtype with 75% A5 (Figure 5C), the production pattern was clearly changed towards a higher DP.

*C. glutamicum* expressing NodC_R346S produces almost exclusively A6, making up 98% of the total oligomers, with A5 accounting for the remaining share (Figure 7D). The product range is relatively stable over the course of the experiment, with only minor changes in the range of 1%–2%. Thus, the amount of hexamer was increased by nearly a quarter.

Clearly, in both expression hosts, the R346S mutein display a similar trend of increased chain length compared to the wildtype.

#### 3.3.3 Yield

**FIGURE 8:**
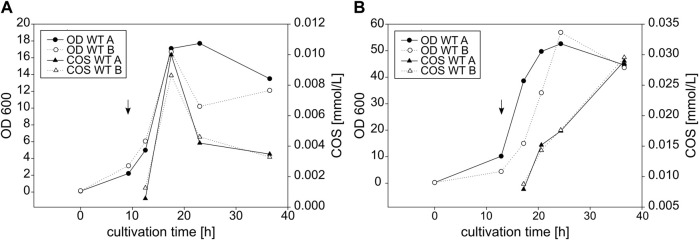
Growth and COS yield of *E coli* **(A)** and *C. glutamicum* **(B)** expressing wildtype NodC_GRH2. Shown is the growth represented by the OD600 and the development of the total COS concentration [mmol/L] over the cultivation time [h]. Depicted as an example are two wildtype cultivations per expression host with the induction indicated by an arrow.

All strains synthesized oligomers with rather consistent product compositions. The yields, however, varied between the expression hosts and, though to a smaller extent, also between the single cultivations of a given strain. As an example, the total COS yields of two individual cultivations each for both *E. coli* and *C. glutamicum* with the wildtype NodC_GRH2 are shown in Figure 8.

For both expression hosts, the COS production clearly is in line with bacterial growth. The protein expression of all cultures was induced at the beginning of their respective exponential phase and only then, they started with COS synthesis. The product concentration rose with increasing OD600; hence, COS production was highest during the exponential growth phase and stagnated (at later timepoints for *C. glutamicum,* see Supplementary Figure S2) or decreased (*E. coli,* Figure 8A) during the stationary phase. While for *E. coli,* product concentration and OD600 develop in parallel, the slope of product formation is delayed for *C. glutamicum,* and the maximum is not reached at the beginning of the stationary phase but rather during it.

For *E. coli*, typical yields of 0.01 mmol/L COS were obtained, but the results were not completely uniform, as higher concentrations of up to 0.05 mmol/L were also reached with the same strain in one experiment (not shown). *C. glutamicum* produced 0.03 mmol/L, but again yields as high as 0.1 mmol/L were also obtained with the wildtype NodC_GRH2 (not shown).

The introduction of the two mutations did not alter the growth of *E. coli* or *C. glutamicum* considerably (see Supplementary Figure S3 in comparison to Figure 8). For both expression hosts, the amount of produced COS was roughly estimated half with the NodC_GRH2 S19L mutein compared to the wildtype, but—as for the wildtype—with strong variation between different cultivations. In contrast, the COS yield with the NodC_GRH2 R346S mutein was comparable to that of the wildtype in *E. coli* and even slightly higher than that of the wildtype in *C. glutamicum*.

## 4 Discussion

### 4.1 *In silico* work

#### 4.1.1 Evaluation of the homology models

The generation of *in silico* models for NodC from *S. meliloti* and *Rhizobium* GRH2 based on an alignment with a bacterial cellulose synthase subunit (BcsA) was successful. While the cytosolic part of the enzyme contains conserved motifs and is thus similar to other crystallized glycosyltransferases, no closely related crystallized template was available for the transmembrane part (Figure 2). Even though crystal structures for two chitin synthases were recently published (Chen et al., 2022; Ren et al., 2022), this new data could not improve our model: both chitin synthases show an even lower sequence identity to NodC_Sm and NodC_GRH2 than the cellulose synthase BcsA (see Supplementary Table S1), and while the nine motifs discussed by Chen et al. are strongly conserved between the chitin polymer synthases of oomycetes, fungi, and arthropods, this applies to a lesser extent only for the rhizobial NodC chitin oligomer synthases. Especially the transmembrane parts of NodCs and the chitin polymer synthases differ from each other, as shown representatively for the chitin synthase from *Candida albicans* and NodC_GRH2 and NodC_Sm in Supplementary Figure S4. This is not surprising given that the transmembrane part of the chitin polymer synthases builds a tunnel to allow secretion of the nascent chitin polymer chain, while NodCs produce chitin oligomers intracellularly and their transmembrane domain possibly serves subcellular localization of the enzymes only. Therefore, we decided to solely rely on the experimentally verified data of Dorfmueller et al. (2014) as the best current model for rhizobial NodCs.

When starting with the NodC_GRH2 sequence only, dependent on the server used for protein prediction, either just the cytosolic part was modelled [SWISS-MODEL (Guex et al., 2009; Bertoni et al., 2017; Bienert et al., 2017; Waterhouse et al., 2018; Studer et al., 2020)] or proteins with less reasonable conformations and unlikely features [I-TASSER (Roy et al., 2010; Yang et al., 2014; Yang and Zhang, 2015); RaptorX (Ma et al., 2015; Wang et al., 2016, 2017); AlphaFold (Jumper et al., 2021)] were generated.

Therefore, the “target-template-alignment” (Guex et al., 2009; Benkert et al., 2011; Mariani et al., 2013; Bienert et al., 2017; Waterhouse et al., 2018; Studer et al., 2020) with the cellulose synthase still seems to be a reasonable approach. Yet, despite a generally homologous structure, there are obvious differences between BcsA and NodC. These include the open tunnel of the cellulose synthase compared to the cleft-like structure of NodC or the difference in size between glucose and GlcNAc. Furthermore, with 3.25 Å (Morgan et al., 2013), the resolution of the template, i.e., the cellulose synthase crystal structure, is rather low (Cohen et al., 2009; Zardecki et al., 2022). At this resolution, usually only the basic scaffolding, the backbone and bulky sidechains can be assigned with a high degree of certainty, but not the atomic structure (Martz et al., 2014; Zardecki et al., 2022).

Still, our model supports the previously published hypothesis (Dorfmueller et al., 2014) and fits our experimental data: the potentially relevant amino acids identified *in silico* actually play an important role in determining COS length *in vitro*, as show below.

#### 4.1.2 Molecular dynamics simulations

As NodC is membrane-embedded (Barny et al., 1996; Dorfmueller et al., 2014), the model’s environment was adjusted for the simulations. The enzyme’s position is derived from experimental data (Dorfmueller et al., 2014), but the flexibility and composition of the *in silico* membrane may not properly represent reality. Lacking experimental data, neither the position of the whole COS nor the orientation of the *N*-acetyl groups is fully validated. The COS can move, but full rotations of the strand, which are e.g., expected to happen during COS elongation (Dorfmueller et al., 2014; Orlean and Funai, 2019) could not be observed. Still, a stable positioning of the units during the simulations tends to indicate correct COS positioning.

Yet, the triplicates of each simulation progressed differently. The outcome was often determined at the beginning of each run, possibly due to the many players involved in a simulation of an oligomer in a membrane-embedded protein. For example, for NodC_GRH2 with the pentamer, the R346 either found S19 at the start of the simulation and built a strong bond, or it engaged with the COS in a less stable interaction. Once the arginine found an interaction partner, a rearrangement became highly unlikely. This is likely an artefact of the simulation.

Still, some frames give a good explanation for the experimental data: for the wildtype NodC_GRH2 with the pentamer, S19 and R346 are close (Table 2) and form hydrogen bonds over considerable parts of the simulation, thereby corroborating the hypothesis of them limiting the depth of the cleft (Dorfmueller et al., 2014). NodC_GRH2 is known as a producer of pentamers in its original organism, the Gram-negative *Rhizobia* (Lopez-Lara et al., 1995), just as when expressed heterologously in *E. coli* (Hamer, 2014). Yet, the synthase can also produce larger COS, which might then be stabilized by R346 or induced by high UDP-GlcNAc pools (Orlean and Funai, 2019). Our experimental results showed an increased proportion of A6 once the bulky arginine was removed, further supporting that this amino acid restricts the products’ length.

For NodC_Sm, the fitting of A6 requires a different rotamer of R349 to give more space first. In Figure 4A, there is clearly enough space for a fifth GlcNAc unit (7.2 Å between R349 and the COS), but not a sixth. This is in line with the reported production (Ardourel et al., 1994; Kamst et al., 2000) and fits our observations: the first mutein (NodC_S19L), which is more similar to NodC_Sm than the wildtype, produces drastically less hexamer (*C. glutamicum* NodC_S19L) or none at all (*E. coli* NodC_S19L).

Further interesting observations from the simulations were an unexpected hydrophobic interaction between L19 and I348 in NodC_Sm. These two are closer in NodC_Sm than R349 and L19 (Table 2) and seem to link two helices (TM1 and TM3, Figure 5). Thus, one might assume that L19 and I348 in NodC_Sm are the true equivalents of S19 and R346 in NodC_GRH2. In contrast, the corresponding I345 in NodC_GRH2 is probably not involved in any interactions with the substrate as it is located on the outside surface of the protein, pointing towards the membrane.

However, this hydrophobic interaction does not seem to be the factor limiting the COS length for NodC_Sm, which is rather brought about by the bulky arginine which is “unoccupied”. In NodC_GRH2, the corresponding amino acid is interacting with S19 by forming H-bonds, thus its position is rather fixed. In NodC_Sm, the arginine is without a partner and can move down the cleft, thereby limiting the room for COS elongation.

In conclusion and in line with the initial hypothesis, the simulations show for NodC_Sm and NodC_GRH2 that the arginine is particularly relevant for COS length: it either interacts with S19, then its position is quite fixed which gives room for COS, or it is more flexible and reduces the depth of the tunnel. Thus, once this amino acid is removed, larger DPs can be synthesized. In addition, I348 seems to play an important role in NodC_Sm as an interaction partner for L19.

### 4.2 *In vitro* work

#### 4.2.1 Evaluation of *E. coli* and *C. glutamicum*

##### 4.2.1.1 Product range

The heterologous expression of rhizobial NodC_GRH2 in both *E. coli* and *C. glutamicum* was successful, all recombinant strains produced COS. To our knowledge, this is the first report of biotechnological COS production using the whole cell factory approach in a bacterial production species other than *E. coli* (Kamst et al., 1997; Zhang et al., 2007).

This study does not consider different protein expression profiles or replication rates of the constructs between the two expression systems. Despite using the same shuttle vector and similar cultivation conditions, the origins of replication [based on pBL1 for *C. glutamicum* and ColE1 for *E. coli* (Eikmanns et al., 1991)] and the sensitivity to IPTG differ (Kortmann et al., 2015; Gomes et al., 2020). For the sake of comparability, only one inducer concentration was applied, yet optimization of this parameter is likely to reduce stress (Dvorak et al., 2015; Gomes et al., 2020) and, therefore, to increase yields.

A first finding was the different range of products between the two expression systems: while wildtype NodC_GRH2 in *E. coli* produces, as expected (Lopez-Lara et al., 1995), mainly pentamer (75%), the same vector construct resulted in 75% hexamer in *C. glutamicum*.

In yeast, low UDP-GlcNAc concentrations—the direct donor substrate for COS synthesis (Zhang et al., 2007)—result in shorter chitin chains (Orlean and Funai, 2019). Likewise, the shorter oligomers in *E. coli* could result from lower intracellular UDP-GlcNAc concentrations compared to *C. glutamicum*. Support for this hypothesis comes from the inherent characteristics of the two species: while *E. coli* is Gram-negative, *C. glutamicum* is a Gram-positive bacterium. As GlcNAc is a vital component of peptidoglycan, a major constituent of Gram-positive cell walls (Typas et al., 2012; Johnson et al., 2013), a higher UDP-GlcNAc pool is likely for *C. glutamicum*. A previous study found an increase of intracellular UDP-GlcNAc levels as well as an increased COS production in *E. coli* after addition of yeast extract to the medium (Zhang et al., 2007). As *E. coli* has a specific transport system for GlcNAc (NagE) and genes relevant for GlcN uptake (*manXYZ*) (Rogers et al., 1988; Peri and Waygood, 1988; Coussement et al., 2020; Vortmann et al., 2021), external addition of GlcNAc and GlcN can help overcome limitations.

In addition, when overexpressing one or several genes for GlcNAc synthesis—*glmS, glmM,* and *glmU* (Coussement et al., 2020)—either the production of hyaluronan, a copolymer of glucuronic acid and GlcNAc, was increased nearly threefold (in *E. coli*) (Woo et al., 2019) or the UDP-GlcNAc pool was increased by a factor of four (in *Lactobacillus casei*) (Rodriguez-Diaz et al., 2012). According to a recent study, the bottleneck of the hexosamine pathway from fructose-6-phosphate (Fru6P) to UDP-GlcNAc in *E. coli* is the first step, the transamination reaction (Coussement et al., 2020). Therefore, this step represents a promising candidate for metabolic engineering to improve COS production.

##### 4.2.1.2 Yield and degradation of COS in the stationary phase

The production of COS was consistent, but the overall yield was relatively low. Yet, the batch cultivations were not performed under controlled conditions; the cells might have suffered from oxygen, carbon, or nutrient limitation or from an acidified medium as these parameters were not monitored, but can be detrimental (Limberg et al., 2017; Valdez-Cruz et al., 2017; Gamboa-Suasnavart et al., 2018; Chopda et al., 2020).

In our experiments, yield was generally higher with *C. glutamicum* compared to *E. coli*. However, in a controlled environment, the yield can most likely easily be increased to an industrial level for both strains. Previous studies on heterologous expression of NodC in *E. coli* obtained gram scale yields (Samain et al., 1997, Samain et al., 1999) and a twenty fold increase in efficiency of the biotechnological production (Moerschbacher, 2017).

Noteworthy is the product decrease in *E. coli* cultivations over time. As the presence of an endochitinase (Francetic et al., 2000) as well as chitin deacetylases (Verma and Mahadevan, 2012) in *E. coli* is known, and growth on GlcN, GlcNAc, and GlcNAc dimer was observed previously (Plumbridge and Pellegrini, 2004; Vortmann et al., 2021), degradation of COS in need for carbon and nitrogen is likely. In contrast, wildtype *C. glutamicum* naturally cannot take up and metabolize GlcNAc (Uhde et al., 2013; Matano et al., 2014).

However, as today’s toolbox for genetic engineering of both *E. coli* and *C. glutamicum* offers plenty of possibilities, uptake or degradation of COS could easily be eliminated. Regarding media optimization, its composition can influence the final products: for *E. coli,* it was shown that modified sugars are incorporated (Zhang et al., 2007).

In summary, testing metabolically engineered strains and/or optimization of the media and cultivation conditions can be expected to increase both, COS length and COS yield.

#### 4.2.2 Production with the WT/muteins

Engineering of NodC_GRH2 to produce certain COS was successful. *E. coli* produces mainly pentamers with the wildtype protein, but roughly 25% consist of a varying mixture composed of A3, A4, and A6. *C. glutamicum* is more consistent with 75% A6 and 25% A5 over the course of the cultivation. The downsizing mutation (S19L) changed the production range for both hosts, but more severely for *C. glutamicum*. Yet, the range of COS also became more uniform for *E. coli*.

In general, the product range is quite consistent for all strains tested. The strongest variations can be observed between the earliest and later time points. At the beginning, the rarer COS might still be below the detection limit. Therefore, later timepoints are more reliable regarding product composition. Still, one can influence the outcome by prematurely terminating the fermentation, as the product range differs, e.g., between the exponential and stationary phase.

Synthesis of longer COS (R346S mutation) was most successful for *C. glutamicum* which produced almost exclusively A6, but also for *E. coli* whose main product was approx. 85% A6. While the production of tetramers only was not achieved, strains synthesizing either mainly pentamers (*E. coli* NodC_GRH2 or the NodC_S19L muteins with both expression systems) or nearly pure hexamers (*C. glutamicum* NodC_R346S) were successfully generated. Especially the practically pure hexamer is interesting for several applications: Firstly, purification is simpler if the synthesized COS are uniform, and A6 is still water-soluble, making it industrially manageable and applicable (Bonin et al., 2020; Chapelle et al., 2021). Secondly, A6 is promising for several biological applications, e.g., plant strengthening (Gubaeva et al., 2018; Shi et al., 2019) or biomedical and pharmaceutical applications (Panda et al., 2012; Zhu et al., 2020).

To broaden the product range, the implementation of other *nod* genes (Mergaert et al., 1995; Samain et al., 1999) or the addition of different chitin deacetylases is also feasible (Hamer et al., 2015). Thereby, the cell factory approach might directly provide defined paCOS with known PA and/or other decorations.

### 5 Conclusion

First, the implementation of COS production in a whole cell factory approach was successful in two different bacterial production species. Second, the product range of the wildtype NodC_GRH2 was as expected for *E. coli*, but *C. glutamicum* synthesized larger oligomers, probably due to its higher UDP-GlcNAc pool as a Gram-positive bacterium (Table 3). Accordingly, the wildtype NodC_GRH2 yielded mainly A5 with *E. coli* and A6 with *C. glutamicum*. For the wildtype, MD simulations showed an interaction of S19 with R346 which stabilizes the arginine’s position at the end of the tunnel. Further, the experiments demonstrated that single amino acid exchanges can direct the production towards smaller/larger oligomers: In the S19L mutein, R346 has no interaction partner, thus it blocks product binding sites which results in a lower DP. For the second mutation, R346S, the bulky arginine is replaced with a small serine creating more room for larger COS and increasing the DP.

**TABLE 3:** Summary of the results. Given is the product range for each cultivation of *E. coli/C. glutamicum* expressing the wildtype or the mutein enzymes as well as a possible explanation.

NodC_GRH2 genotype	*E. coli*	*C.* *glutamicum*	Explanation
**WT**	**A4 (19%)**	A4 (1%)	Expression host influence: Higher UDP-GlcNAc pool in Gram-positive bacteria → longer COS with *C. glutamicum*
	**A5 (75%)**	**A5 (25%)**	S19 and R346 interact → A5 main product in *E. coli*
	A6 (6%)	**A6 (75%)**	
**S19L** DP↓	**A4 (30%)**	A4 (3%)	“Free” R346 (no interaction with L19) → R346 protrudes into the channel → less room for COS → DP decreases
	**A5 (70%)**	**A5 (54%) **	
	–	**A6 (44%)**	
**R346S** DP↑	A4 (9%)	–	Bulky R346 is exchanged with small serine → more space for longer COS → DP increases
	A5 (6%)	A5 (2%)	
	**A6 (86%)**	**A6 (98%)**	

#### 5.1 Which strain is better for chitooligosaccharides synthesis?

As mentioned before, the COS composition is broader with *E. coli* (*E. coli* WT, NodC_S19L) compared to the more uniform product range of *C. glutamicum* (esp. NodC_R346S, Table 3)*,* with the latter facilitating subsequent purification*.* Moreover, the obtained oligomers were not only longer, but the yield was also higher for *C. glutamicum*. One further drawback of COS production with *E. coli* could be the production of endotoxins, which makes them unsuitable for certain applications (Lieder et al., 2013), or requires costly removal (de Vries et al., 2018). In contrast, *C. glutamicum* does not produce endotoxins, is non-sporulating, and generally regarded as safe (GRAS) (Blombach and Eikmanns, 2014; Lee et al., 2016). Unlike *E. coli, C. glutamicum* shows only weak catabolite repression (Baritugo et al., 2018) and cannot metabolize COS naturally (Uhde et al., 2013; Matano et al., 2014)*.*

In summary, *C. glutamicum* is a more promising candidate for COS production. As a known workhorse from industrial applications, biotechnological tools for modification, knowledge about metabolic pathways, and efficient cultivation systems are readily available (Lee et al., 2016; Baritugo et al., 2018). Moreover, with a possible addition of further genes modifying COS, the product range can be expanded, potentially allowing cost-effective large-scale production of customized oligomers.

## Data Availability

Representative frames from the MD simulations including the protein models of NodC_GRH2 and NodC_Sm with their natural substrates can be found in the repository of Münster University, Miami (https://miami.uni-muenster.de/) https://doi.org/10.17879/74089643745.
