# Supplementary material for: Customized chitooligosaccharide production—controlling their length via engineering of rhizobial chitin synthases and the choice of expression system
Source: Front Bioeng Biotechnol. 2022 Dec 14;10:1073447. doi: 10.3389/fbioe.2022.1073447 (PMC9795070; doi:10.3389/fbioe.2022.1073447)
Supplement: Supplementary file 1 [file DataSheet1.pdf]

Supplementary Material

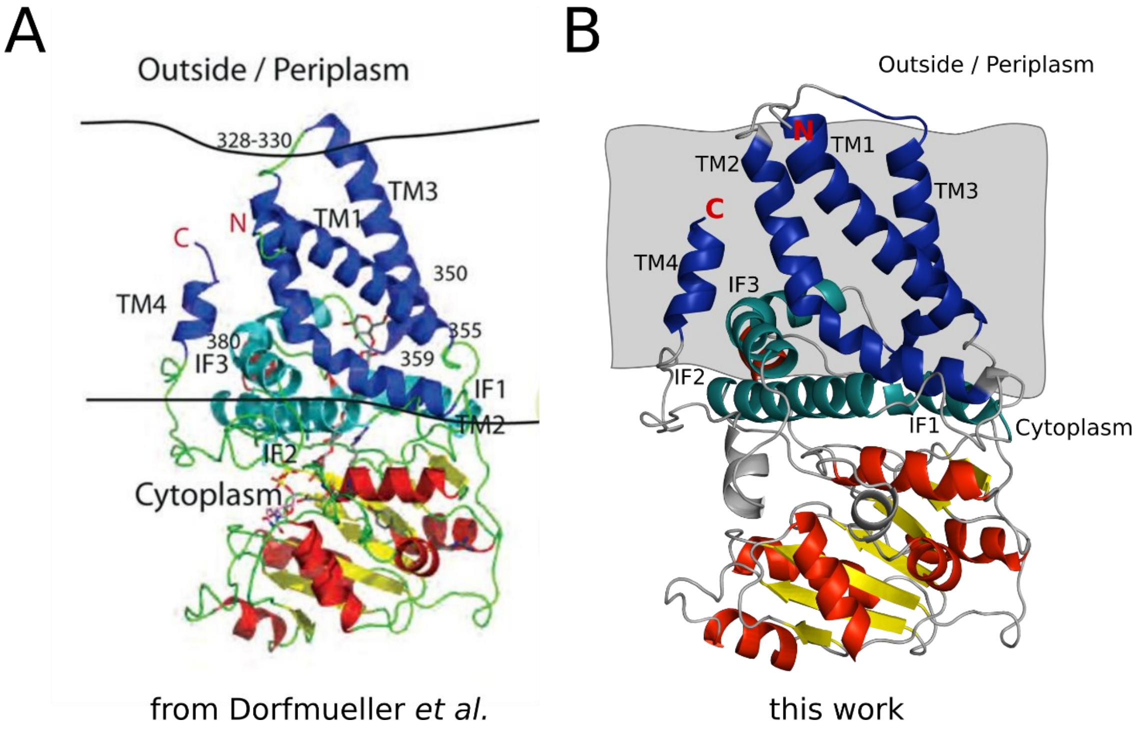

Supplementary Figure 1: Structural NodC_Sm models. Depicted are (A) the model published by Dorfmueller *et al*. (Dorfmueller et al., 2014) and (B) the model created in this work.

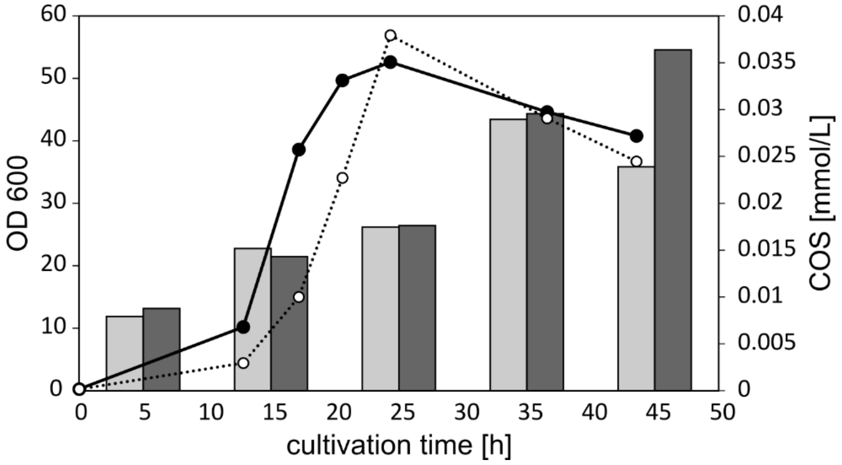

Supplementary Figure 2: *C. glutamicum* yield and growth with NodC_GRH2 wildtype. Depicted is the growth measured via the optical density (OD600, lines) and the COS concentration [mmol/L] (bars) over time for two exemplary cultivations (light and dark). The amount of product increases throughout the cultivation.

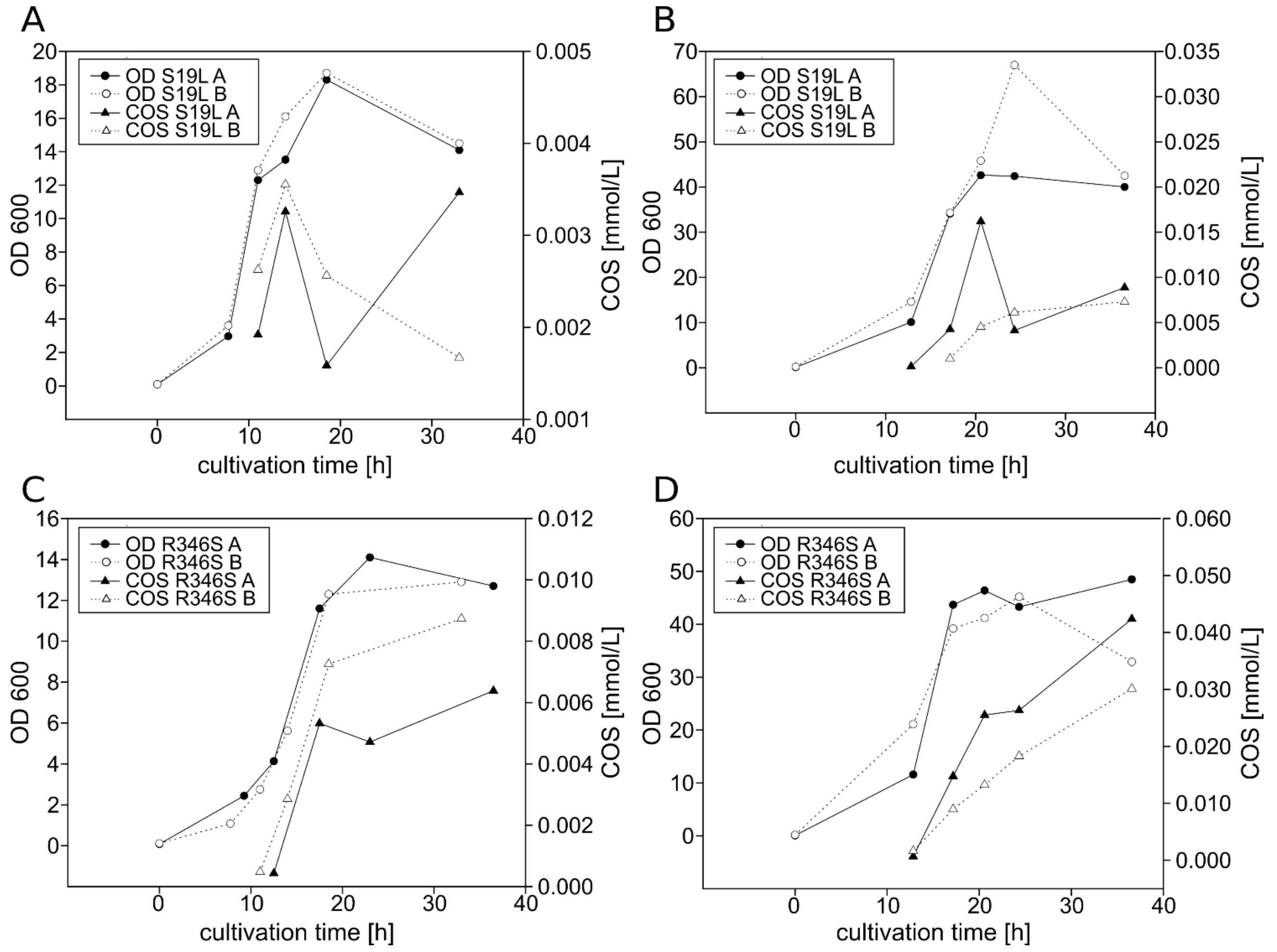

Supplementary Figure 3: Growth and COS yield of *E. coli* (left, A+C) and *C. glutamicum* (right, B+D) expressing the NodC_GRH2 muteins S19L (top, A+B) or R346S (bottom, C+D). Shown is the growth represented by the OD600 and the development of the total COS concentration [mmol/L] over the cultivation time [h]. Depicted as an example are different two cultivations per expression host per mutein.

Supplementary Table 1: Sequence identity matrix. Shown is the sequence identity based on a multiple sequence alignment with MUSCLE (Edgar, 2004) between the amino acid sequences of NodC_GRH2 (UniProt: A0A0N7ARR3) and NodC_Sm (Gene ID at NCBI: 61599275) and the amino acid sequences of three potential modelling templates with available crystal structures: chitin synthase from *Candida albicans* (PDB: 7STL_A; Ren et al., 2022), chitin synthase from *Phytophthora sojae* (Protein ID at NCBI: XP_009524159, PDB: 7WJM_A; Chen et al., 2022) and cellulose synthase from *Rhodobacter sphaeroides* (PDB: 4HG6_A).

	**chitin synthase *Candida albicans***	**chitin synthase *Phytophthora sojae***	**cellulose synthase *Rhodobacter sphaeroides***	**NodC_GRH2**	**NodC_Sm**
**chitin synthase *Candida albicans***	100.00				
**chitin synthase *Phytophthora sojae***	29.58	100.00			
**cellulose synthase *Rhodobacter sphaeroides***	15.63	15.85	100.00		
**NodC_GRH2**	18.37	21.53	22.97	100.00	
**NodC_Sm**	20.99	22.41	25.73	69.50	100.00

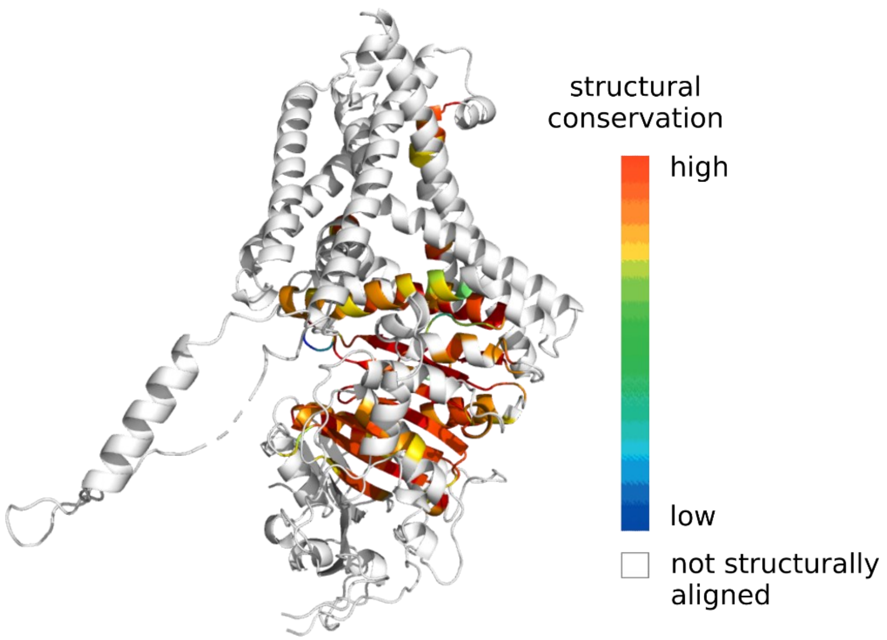

Supplementary Figure 4: Fungal chitin synthase crystal structure (7STL_A). The coloring represents the structural conservation between the chitin synthase from *Candida albicans* (PDB: 7STL_A; Ren et al., 2022) and our models for NodC_GRH2 and NodC_Sm calculated using the PyMOL plugin PyMod 3.0.1 (Janson and Paiardini, 2021) with the structural alignment tool SALIGN and the coloring based on structural conservation (SCR_FIND) with a SC-score limit of 1.0.

Chen, W., Cao, P., Liu, Y., Yu, A., Wang, D., Chen, L., et al. (2022). Structural basis for directional chitin biosynthesis. *Nature*. doi: 10.1038/s41586-022-05244-5.

Dorfmueller, H. C., Ferenbach, A. T., Borodkin, V. S., and Van Aalten, D. M. F. (2014). A structural and biochemical model of processive chitin synthesis. *J. Biol. Chem.* 289, 23020–23028. doi: 10.1074/jbc.M114.563353.

Edgar, R. C. (2004). MUSCLE: Multiple sequence alignment with high accuracy and high throughput. *Nucleic Acids Res.* 32, 1792–1797. doi: 10.1093/nar/gkh340.

Janson, G., and Paiardini, A. (2021). PyMod 3: A complete suite for structural bioinformatics in PyMOL. *Bioinformatics* 37, 1471–1472. doi: 10.1093/bioinformatics/btaa849.

Ren, Z., Chhetri, A., Guan, Z., Suo, Y., Yokoyama, K., and Lee, S. (2022). Structural basis for inhibition and regulation of a chitin synthase from Candida albicans. 29. doi: 10.1038/s41594-022-00791-x.
